# The Kallikrein–Kinin System: Proteolytic Orchestrators of Tissue Barrier Disruption in Inflammation and Cancer

**DOI:** 10.3390/ijms27167282

**Published:** 2026-08-15

**Authors:** Areli Cárdenas-Oyarzo, Carlos D. Figueroa, Ricardo Huilcamán, Larissa Turones, Sergio Martínez-Huenchullán, Pamela Ehrenfeld

**Affiliations:** 1 Centro Integrativo de Biología y Química Aplicada (CIBQA), Facultad de Ciencias de la Salud, Universidad Bernardo OHiggins, General Gana 1702, Santiago 8370854, Chile; arelic@docente.ubo.cl; 2 Laboratory of Cellular Pathology, Institute of Anatomy, Histology & Pathology, Faculty of Medicine, Universidad Austral de Chile, Valdivia 5090000, Chile; cdfigueroa@gmail.com (C.D.F.); larissa.cordova@uach.cl (L.T.); 3 The Kallikrein House, Universidad Austral de Chile, Valdivia 5090000, Chile; 4 School of Medical Technology, Faculty of Health Sciences, Universidad Bernardo O’Higgins, Santiago 8370854, Chile; ricardo.huilcaman@ubo.cl; 5 Center for Interdisciplinary Studies on the Nervous System (CISNe), Universidad Austral de Chile, Valdivia 5090000, Chile; 6 School of Physical Therapy, Universidad San Sebastián, Valdivia 5090000, Chile; sergio.martinez@uss.cl

**Keywords:** kinin peptides, kallikrein-related peptidases, cell adhesion, kinin receptors, inflammation, cancer

## Abstract

The kallikrein–kinin system (KKS) and the kallikrein-related peptidase (KLK) family are interconnected proteolytic networks that regulate inflammatory signaling, vascular permeability, extracellular matrix remodeling, and tissue barrier dynamics. Beyond their classical vasoactive and inflammatory functions, accumulating evidence indicates that kinin peptides, including bradykinin, Lys-bradykinin, and their des-Arg^9^ metabolites, together with selected KLKs, modulate cell–cell and cell–extracellular matrix adhesion. Through B1 and B2 kinin receptor activation, the KKS influences endothelial adhesion molecule expression, leukocyte integrin activation, neutrophil trafficking, focal adhesion kinase/Src signaling, cytoskeletal remodeling, and matrix metalloproteinase activity. In parallel, KLKs directly reshape the adhesive microenvironment by cleaving junctional proteins, including E-cadherin and desmosomal components, and extracellular matrix substrates such as fibronectin, laminin, vitronectin, fibrinogen, and collagens. These coordinated actions affect epithelial and endothelial barrier integrity, leukocyte transmigration, angiogenesis, fibrosis, epithelial–mesenchymal transition, tumor cell migration, invasion, and metastatic dissemination. This review critically summarizes current evidence linking KKS and KLK activity to adhesion-dependent processes in inflammation and cancer, emphasizing how proteolytic signaling may either preserve tissue homeostasis or promote pathological barrier disruption depending on cellular context, receptor expression, protease activity, and microenvironmental cues. Understanding these mechanisms may refine the identification of adhesion-related biomarkers and support the development of targeted therapeutic strategies for inflammatory disorders, fibrotic remodeling, and cancer progression.

## 1. Introduction

Cell adhesion is a highly regulated process required for homeostasis, tissue organization, barrier integrity, and immune surveillance. From the earliest stages of embryonic development, interactions between cells and their microenvironment are essential for tissue architecture and function. These interactions are primarily organized through two major adhesion modalities: cell–cell adhesion and cell–extracellular matrix (cell–ECM) adhesion.

Cell–cell adhesion enables physical and biochemical communication between adjacent cells and contributes to the establishment of protective epithelial and endothelial barriers. This process is highly dynamic during embryonic development, epithelial proliferation, tissue repair, and inflammatory responses, including neutrophil adhesion to the microvascular endothelium [1,2]. In parallel, cell–ECM adhesion mediates cellular attachment to the ECM’s structural components and is largely mediated by focal adhesions, which are dynamic organelles linked to the actin cytoskeleton. Through the integration of integrins, adaptor proteins, and signaling molecules, focal adhesions regulate cytoskeletal organization, cell migration, and survival [3,4].

Several proteolytic and signaling pathways regulate both cell–cell and cell–ECM adhesion, including the kallikrein–kinin system (KKS) and kallikrein-related peptidases (KLKs). The KKS is a proteolytic cascade classically involved in inflammation, vasodilation, vascular permeability, and pain. In parallel, KLKs mediate extracellular proteolysis and tissue remodeling. Increasing evidence indicates that both systems modulate endothelial behavior, leukocyte recruitment, angiogenesis, ECM organization, and adhesion-dependent signaling [5].

**Figure 1:**
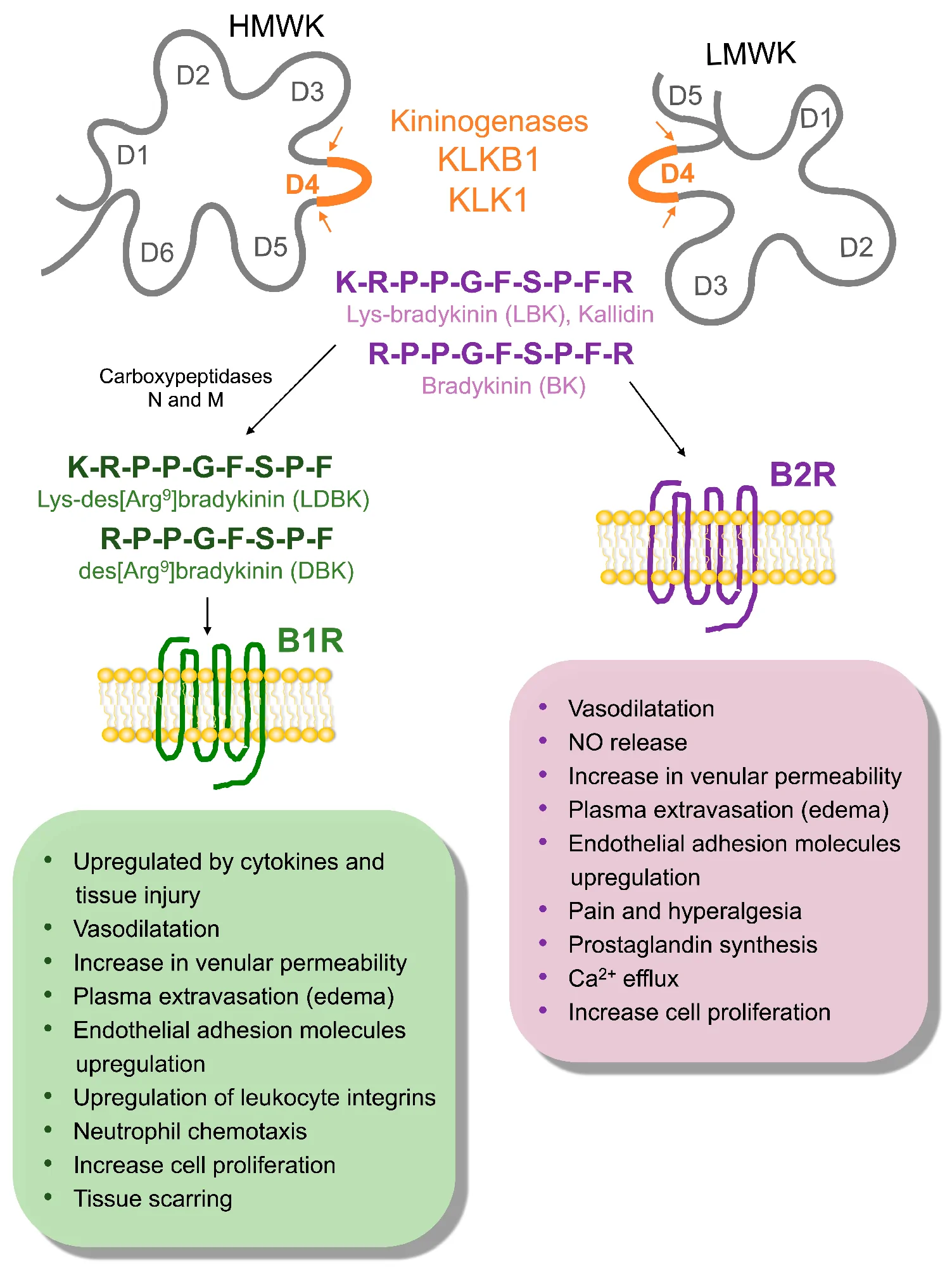
Generation of kinin peptides from kininogen precursors and their receptor selectivity. High-molecular-weight kininogen (HMWK) and low-molecular-weight kininogen (LMWK) are structurally organized into multiple domains (D1–D6 and D1–D5, respectively), with the kinin moiety embedded within domain D4 (highlighted in orange). Kininogenases encoded by KLKB1 (plasma kallikrein) and KLK1 (tissue kallikrein) cleave within D4 to release Lys-bradykinin (LBK/kallidin; K-R-P-P-G-F-S-P-F-R) from HMWK and bradykinin (BK; R-P-P-G-F-S-P-F-R) from both HMWK and LMWK, respectively. Subsequent removal of the C-terminal arginine residue by carboxypeptidases N and M generates des[Arg^9^]-bradykinin (DBK; R-P-P-G-F-S-P-F) and Lys-des[Arg^9^]-bradykinin (LDBK; K-R-P-P-G-F-S-P-F). BK and LBK preferentially activate the constitutively expressed kinin B2 receptor (B2R), whereas DBK and LDBK preferentially activate the inducible kinin B1 receptor (B1R). B2R activation mediates vasodilation, nitric oxide (NO) release, increased venular permeability, plasma extravasation (edema), upregulation of endothelial adhesion molecules, pain and hyperalgesia, prostaglandin synthesis, Ca^2+^ efflux, and increased cell proliferation. B1R is upregulated by cytokines and tissue injury and mediates vasodilation, increased venular permeability, plasma extravasation (edema), upregulation of endothelial adhesion molecules, upregulation of leukocyte integrins, neutrophil chemotaxis, and increased cell proliferation. HMWK: high-molecular-weight kininogen; LMWK: low-molecular-weight kininogen; KLKB1: plasma kallikrein; KLK1: tissue kallikrein; BK: bradykinin; Lys-BK: Lys-bradykinin/kallidin; DBK: des[Arg^9^]-bradykinin; LDBK: Lys-des[Arg^9^]-bradykinin; B1R: kinin B1 receptor; B2R: kinin B2 receptor; NO: nitric oxide.

Kinins are bioactive peptides released from high- and low-molecular-weight kininogens through the enzymatic activity of plasma kallikrein and tissue kallikrein, encoded by the *KLKB1* and *KLK1* genes, respectively. For this reason, these proteases are also known as kininogenases [6,7]. In mammals, KLKB1 and KLK1 proteins generate bradykinin (BK) and Lys-bradykinin/kallidin (LBK), which mainly activate the kinin B2 receptor (BDKR2 gene, B2R). Proteolytic cleavage of the C-terminal arginine residue by carboxypeptidases N and M produces Lys-des[Arg^9^]-bradykinin (LDBK) and des[Arg^9^]-bradykinin (DBK), which preferentially activate the kinin B1 receptor (BDKR1 gene, B1R) [7] (Figure 1).

KLK1 belongs to the kallikrein-related peptidase family (KLK), which comprises 15 members encoded by genes located in tandem on chromosome 19q13.3–q13.4 [8,9,10]. KLKs are expressed in specific cell types and secreted into the extracellular milieu or body fluids. All KLK family members display trypsin- or chymotrypsin-like activity and are generally synthesized as inactive pre-proenzymes. Their activation occurs via proteolytic cascades, either through autoactivation or cleavage by other proteases. Pro-KLK5 autoactivation has been proposed as an initiating event in the KLK activation cascade [11]. This cascade is tightly regulated by endogenous inhibitors encoded by the SPINK gene family, located on chromosome 5q32, which help maintain proteolytic balance under physiological conditions [12,13,14].

Kinin peptides and their receptors regulate adhesion-related processes by modulating integrin expression and activating intracellular signaling molecules such as paxillin and focal adhesion kinase (FAK). In parallel, KLKs contribute to ECM remodeling by cleaving fibronectin and other matrix components, thereby altering the adhesive landscape of tissues [15]. Consequently, dysregulation of KKS and KLK activity may affect physiological and pathological processes that depend on adhesive interactions, including inflammation, vascular permeability, blood coagulation, pain perception, tissue remodeling, and cancer progression [16,17,18].

Given the central role of adhesion in these biological responses, this review discusses the evidence available to date on how KKS and KLK members regulate cell adhesion across diverse cellular contexts, with emphasis on mechanisms involved in tissue homeostasis, barrier disruption, inflammation, and disease progression.

## 2. The KKS and Cell-to-Cell Interaction: Regulation of Junctional Proteins and Leukocyte Behavior

Dynamic changes in cell adhesion are required for tissue organization, renewal, and repair, processes in which the KKS may act as a proteolytic and signaling modulator [2,19]. Cell-to-cell junctions, including tight junctions, adherens junctions, and desmosomes, form extracellular connections between adjacent cells and the cytoskeleton. These structures preserve epithelial integrity, barrier function, and tissue architecture. Disruption of junctional complexes contributes to pathological tissue remodeling. For example, KLK-mediated shedding of the E-cadherin extracellular domain may weaken adherens junctions and facilitate tumor cell dissemination [4,20,21]. Similarly, remodeling of tight junction proteins such as claudin-5, occludin, ZO-1, and members of the junctional adhesion molecule (JAM) family modulates endothelial permeability and paracellular migration [22,23,24]. In inflammatory settings, cell-to-cell adhesion also involves transient interactions between leukocytes and endothelial cells. Increased expression of adhesion molecules such as intercellular adhesion molecule type 1 (ICAM-1) supports leukocyte adhesion, crawling, and transendothelial migration [25,26]. Thus, KKS- and KLK-dependent regulation of junctional and adhesion molecules may contribute to leukocyte extravasation, epithelial plasticity, tumor metastasis, vascular leakage, and fibrosis [2,27].

### 2.1. Kinin Receptors Facilitate Leukocyte–Endothelial Interactions During Inflammation

**Figure 2:**
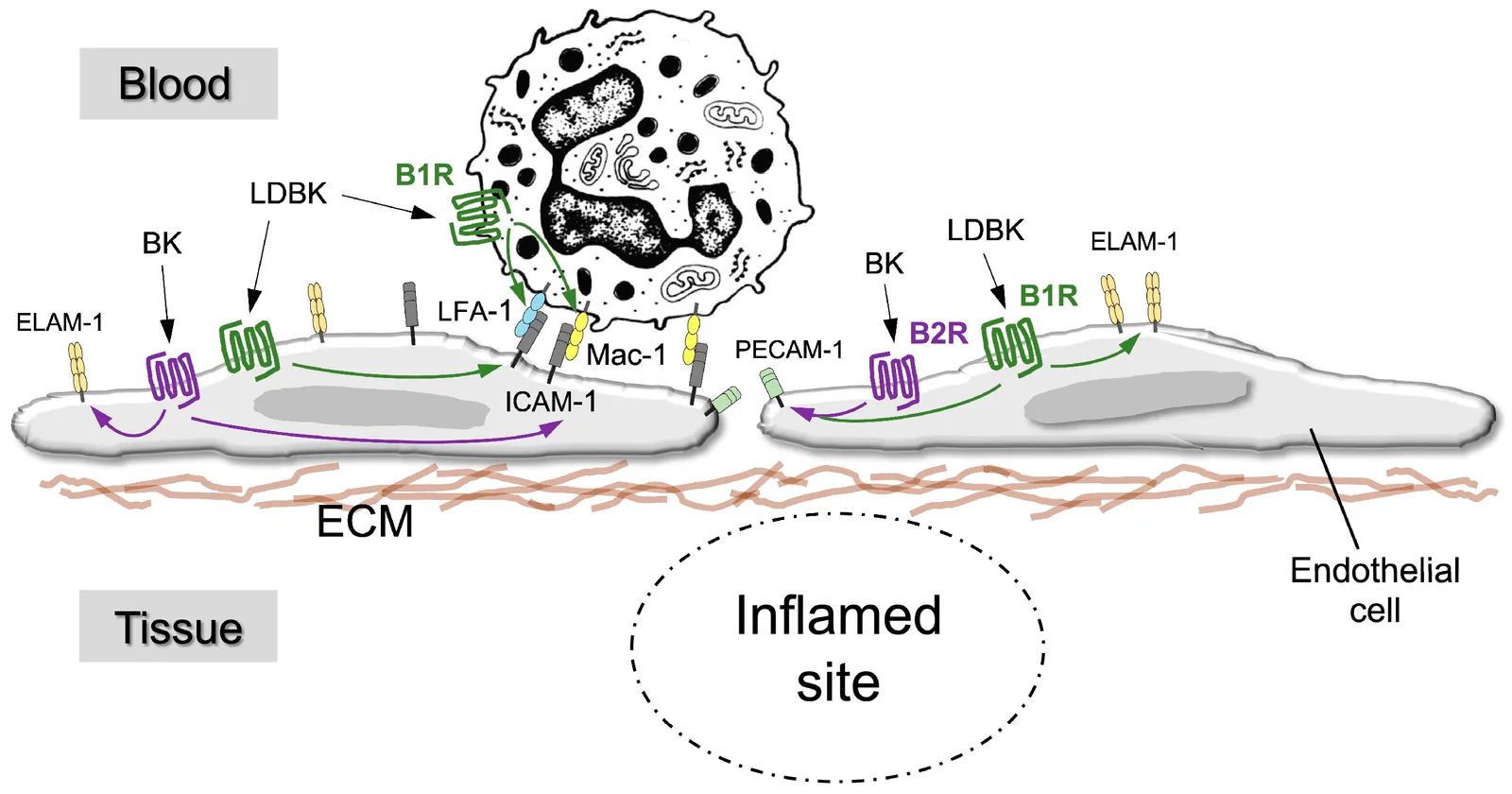
Modulation of leukocyte adhesion and migration under kinin stimulation. Activation of B1R (by LDBK) or B2R (by BK) controls the expression and affinity of endothelial adhesion molecules (ICAM-1; PECAM-1) and integrins (LFA-1, Mac-1) of polymorphonuclear leukocytes (PMN). These changes in both endothelial cells and leukocytes promote rolling, firm adhesion, and subsequent transmigration of immune cells toward inflammation sites. LDBK: Lys-des[Arg^9^]-bradykinin; BK: bradykinin; ICAM-1: intercellular adhesion molecule type 1; PECAM-1: platelet endothelial cell adhesion molecule-1; LFA-1: lymphocyte function-associated antigen-1; Mac-1: macrophage-1 antigen; PMN: polymorphonuclear leukocytes; ELAM-1: endothelial leukocyte adhesion molecule-1.

The KKS contributes to leukocyte recruitment by regulating interactions between immune cells and the vascular endothelium. B1R and B2R signaling modulate leukocyte activation, adhesion, and migration during inflammatory responses [28,29,30]. Neutrophils and monocytes express several KKS components, including KLK1, HMWK, LMWK and kinin-binding sites, supporting local autocrine and paracrine kinin signaling [31,32,33,34,35,36,37,38]. Neutrophils also possess the enzymatic machinery required to generate and respond to kinins. Since the late 1980s, studies have demonstrated the expression and activity of KLK1 in human neutrophils [31] (Figure 2). In addition, radioligand binding assays confirmed the presence of B1R and B2R binding sites in these cells, indicating that neutrophils can respond to locally generated kinin peptides [39,40,41]. These findings support the concept that leukocytes are not merely targets of circulating kinins but active participants in local KKS signaling.

Leukocyte responsiveness to kinins depends strongly on the cell’s activation state. In vivo models using B1R antagonism or genetic deletion have shown reduced recruitment of polymorphonuclear leukocytes (PMNs) to inflammatory sites [29,30]. This response may be amplified locally, as neutrophil activity can promote BK release and the subsequent generation of the B1R agonists DBK and LDBK [39]. Similar kinin-dependent mechanisms have been described in monocytes, macrophages, and lymphocytes, where B1R activation contributes to mediator release and immune cell trafficking [42,43].

The temporal expression of kinin receptors shapes the intensity and duration of the neutrophil response. Consistent with the inducible nature of B1R described above, in neutrophils, IL-1β induces a priming effect that enhances their chemotactic responses to LDBK, more relevant during sustained or chronic inflammation [6,40,44]. Additional mechanisms, including receptor oligomerization or plasma membrane mobilization, may further fine-tune this response. Together, these findings indicate that the KKS integrates biochemical and adhesive signals required for leukocyte–endothelial interactions (Figure 2). Although this section focuses on kinin receptor-dependent leukocyte–endothelial interactions, KLK-mediated proteolysis may also shape immune cell recruitment in tumors. In this context, recent evidence has identified KLK6 as a regulator of neutrophil recruitment and immunosuppressive remodeling in pancreatic cancer models [45]. This finding supports a broader role for kallikrein-related proteases in tumor-associated inflammatory cell trafficking, complementing the KKS-driven mechanisms described above.

#### 2.1.1. Integrin Regulation During Leukocyte Adhesion

Kinin-driven neutrophil trafficking involves transendothelial and/or paracellular migration across the endothelial barrier [6,46,47,48]. Several studies have shown that kinin peptides enhance leukocyte adhesion to ECM proteins and endothelial cells by modulating integrins and adhesion molecules [49]. BK and LDBK increase the expression of Mac-1 and LFA-1 in neutrophils and monocyte-like cells, while also promoting ICAM-1 expression in endothelial cells [49,50]. LFA-1 and Mac-1 mediate distinct but coordinated steps of leukocyte recruitment. LFA-1 contributes to neutrophil rolling, adhesion stabilization, and early migration, whereas Mac-1 supports firm arrest through interaction with ICAM-1 on endothelial cells [51]. These data indicate that kinin signaling strengthens leukocyte–endothelial adhesion by coordinating integrin activation on leukocytes with adhesion molecule expression on endothelial cells (Figure 2).

#### 2.1.2. Proteolytic and Oxidative Remodeling During Leukocyte–Endothelial Interaction

Beyond regulating adhesion molecules, B1R signaling promotes the release of inflammatory and proteolytic mediators. Upon B1R activation, neutrophils release myeloperoxidase (MPO) and matrix metalloproteinase-9 (MMP-9) into the local microenvironment. These mediators may contribute to extracellular matrix remodeling and increased endothelial permeability during inflammation. MPO-dependent oxidative modifications may further alter the adhesive microenvironment. MPO contributes to 3-nitrotyrosine (NO2Tyr) formation, which can accumulate in the ECM at injured sites and promote fibronectin nitration [52]. Whether kinin receptors directly regulate nitration of adhesion molecules remains unclear. However, local kinin formation at the neutrophil surface may contribute to endothelial contraction and plasma exudation during inflammation [49].

#### 2.1.3. Signaling Pathways Controlling Diapedesis and Transmigration

B1R may participate in multiple phases of neutrophil diapedesis, including rolling, firm adhesion, and transmigration. Stimulation of endothelial cells with a B1R agonist increases ELAM-1, which contributes to early neutrophil rolling, as well as ICAM-1 and PECAM-1, which participate in firm adhesion and transmigration [49]. ICAM-1-mediated adhesion may also promote PECAM-1 activation, supporting sequential progression through the transmigration cascade [49,53].

Kinin receptor signaling regulates leukocyte transmigration through pathways involving PKC, PI3K, and MAPKs. These pathways modulate the expression of ICAM-1 and PECAM-1 on endothelial cells and promote cytoskeletal remodeling associated with membrane protrusion formation [49,54,55,56,57]. Thus, kinin receptors may coordinate both adhesive receptor expression and endothelial structural changes required for leukocyte passage [49,58].

The final steps of transmigration require dynamic regulation of leukocyte–endothelial adhesion. LFA-1 controls neutrophil attachment to ICAM-1 and ICAM-2, whereas Mac-1 binds additional ligands, including RAGE, JAM-A, and JAM-C [59]. Whether kinin receptors can mediate these divergent events while still providing the necessary binding forces to direct neutrophil migration remains to be determined. Efficient neutrophil transmigration in inflamed tissue requires dynamic regulation of ICAM-1-mediated adhesion [60]. ICAM-1 cleavage may also facilitate leukocyte detachment from the endothelial surface. In this context, ADAM10-mediated cleavage of ICAM-1 has been proposed to support neutrophil release during the final steps of diapedesis [61]. Whether B1R and/or B2R signaling modulates ADAM10 activity in inflammation and leukocyte transmigration remains unknown.

As a future pharmacological projection, the modulation of CAMs and integrins by kinin ligands has been shown to contribute to inflammatory vascular disorders such as vasculitis, where leukocyte–endothelial interactions play a central pathogenic role [62]. Overall, current evidence supports the participation of the KKS in leukocyte extravasation through coordinated regulation of integrins, CAMs, endothelial signaling, and transmigration pathways (Figure 2).

#### 2.1.4. B1R and B2R Promote Neutrophil Adhesion and Activation in Fibrosis

Beyond acute inflammation, B1R and B2R signaling may contribute to fibrosis by sustaining leukocyte recruitment and chronic inflammatory activation [63,64]. This process involves dysregulated cell adhesion at both cell–cell and cell–matrix interfaces, which progressively disrupts tissue homeostasis [1,2].

Kinin signaling contributes to the release of profibrotic mediators in pulmonary tissues. In alveolar epithelial cells and lung fibroblasts, BK stimulates the secretion of chemotactic and profibrotic mediators, including IL-6, IL-8, MCP-1, and TGF-β [65,66]. These findings suggest that kinin-mediated signaling can coordinate neutrophil and monocyte recruitment while shaping a proinflammatory and profibrotic microenvironment.

Similar mechanisms have been described in liver fibrosis models. In mice, B1R expression increases during fibrogenesis, whereas pharmacological blockade using the selective antagonist BI 113823 attenuates portal hypertension, leukocyte activation, and ECM deposition [63]. This inhibition also reduces the expression of inflammatory cytokines and profibrotic markers, including α-SMA, connective tissue growth factor (CTGF), and collagens I, III, and IV [64].

Although the precise links between kinin receptors, CAM regulation, and fibrosis remain incompletely defined, current evidence supports kinin signaling as a potential therapeutic target to limit chronic inflammation and pathological tissue remodeling.

### 2.2. B2R Regulates Adherens Junction Proteins Under Physiological and Pathological Conditions

B2R regulates junctional proteins in both physiological and pathological contexts. During embryonic development, BK acting through B2R promotes cell–cell adhesion in collecting duct cells by upregulating E-cadherin and recruiting β-catenin to cell–cell contacts, resulting in cell compaction and tissue morphogenesis [67]. These effects depend on PI3K/Akt signaling and are abolished by the B2R antagonist HOE-140 [68].

Under pathological conditions, B2R activation can modulate epithelial and endothelial barrier permeability by altering cell–cell junction organization. In an experimental nephrotoxicity model, B2R activation exerted protective effects by reducing oxidative stress, inflammation, ICAM-1 expression, and TGF-β levels [69]. Conversely, in glioma models, BK increases blood–tumor barrier permeability and decreases the expression of tight junction proteins such as ZO-1, occludin, and claudin-5 [22,70]. In line with this concept, Batista et al. recently showed that B1R signaling can also modulate blood–brain barrier permeability in a glioblastoma-associated microenvironment, extending the role of kinin receptors in barrier dysfunction beyond B2R-dependent mechanisms [71].

BK-induced ZO-1 rearrangement has also been described in mouse podocytes, supporting a broader role for kinin signaling in junctional regulation [72].

**Figure 3:**
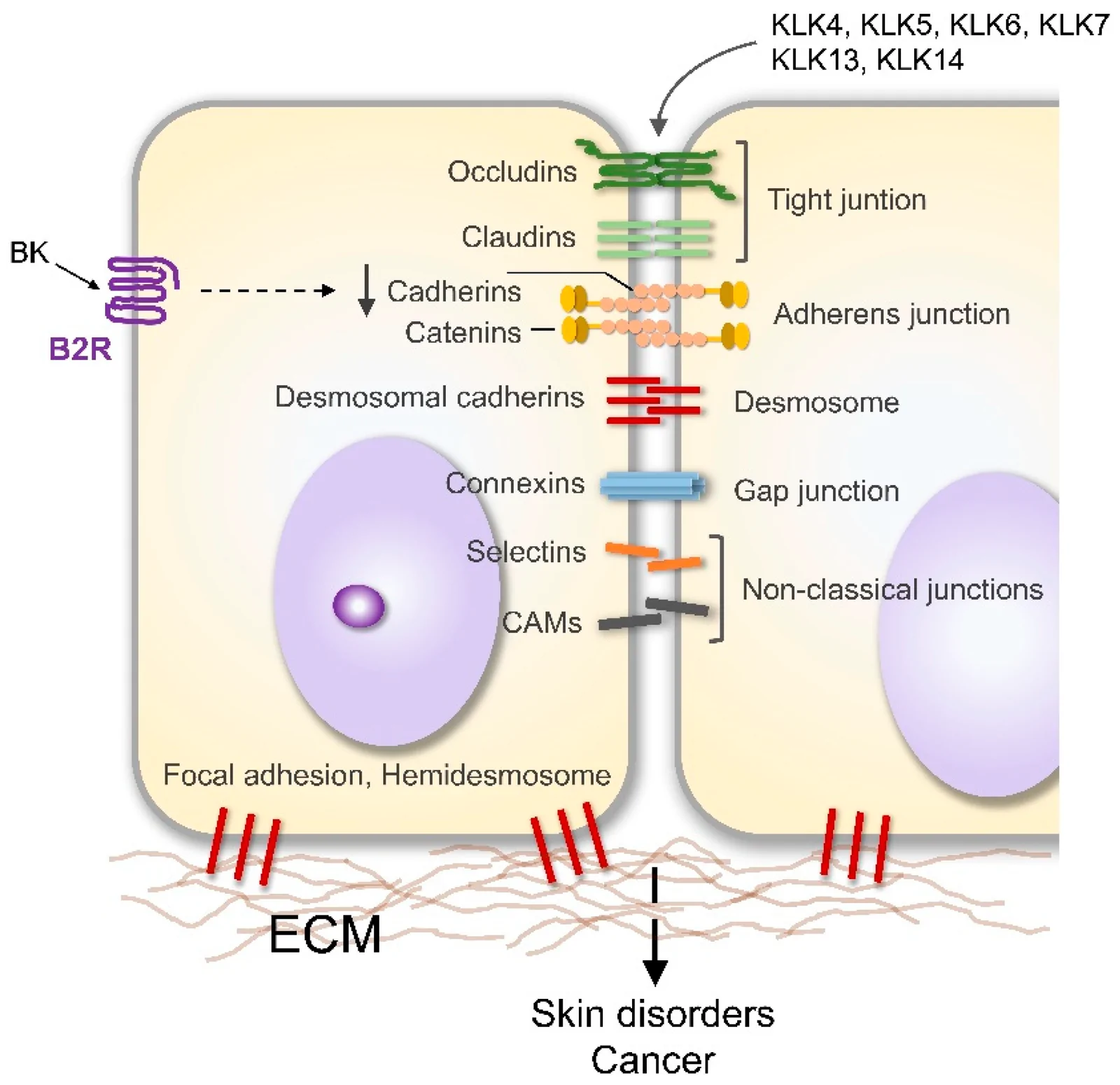
Potential regulation of cell–cell junctions by KLKs and B2R signaling. Selected KLKs, including KLK4–7 and KLK14, and in a context-dependent manner, KLK13, may contribute to the remodeling of intercellular junctions through proteolytic cleavage of adhesion molecules and junctional proteins. Potential targets of KLKs (grey arrow) include components of tight junctions (occludins and claudins), adherens junctions (cadherins and catenins), desmosomes (desmosomal cadherins), and other cell adhesion molecules (CAMs), thereby altering epithelial cohesion and barrier integrity. In parallel, BK acting through the B2R may modulate cadherin-dependent adhesion (indicated by the down grey arrow) and junctional organization. Together, these mechanisms may contribute to epithelial barrier modifications (stability or destabilization), tissue remodeling, and disease progression in inflammatory skin disorders and cancer (as indicated by black arrow at the end of ECM). BK: bradykinin; B2R: kinin B2 receptor; KLKs: kallikrein-related peptidases; CAMs: cell adhesion molecules; ECM: extracellular matrix.

In cancer, B2R signaling may promote invasive behavior through junctional disruption. In gastric cancer, BK/B2R activation enhances migration, invasion, and tumor progression via ERK1/2 activation, MMP-2/9 upregulation, and E-cadherin downregulation [73]. Together, these findings indicate that B2R can support epithelial cohesion during development but contribute to junctional remodeling, permeability, and invasion in pathological contexts (Figure 3).

### 2.3. Kallikrein-Related Peptidases Regulate Cell-to-Cell Adhesion in Cancer

KLKs have emerged as context-dependent modulators of cell–cell adhesion in cancer. Rather than acting only as degradative enzymes, KLKs dynamically reprogram adhesive states by destabilizing stable junctions and promoting migratory cellular phenotypes. Although direct evidence remains limited for some family members, accumulating studies indicate that KLKs can cleave junctional proteins, disrupt epithelial cohesion, remodel the extracellular microenvironment, and promote invasive behavior. These effects are consistent with the roles of serine proteases in cancer progression, including ECM remodeling, dysregulated integrin signaling, EMT-like phenotypes, and metastasis [74].

KLKs were first characterized in skin physiology and desquamation, where KLK5, KLK6, KLK7, KLK8, and KLK14 cleave components of corneodesmosomes to enable epidermal shedding [75,76,77]. Consistent with this role, KLK5 knockout mice showed enhanced desmosomal integrity and resistance to skin tumor development, supporting the involvement of KLK5 in junctional remodeling, epithelial cohesion, and carcinogenesis [78]. These findings provided an early in vivo link between epidermal microstructure and tumorigenesis and supported the concept that desmosomes may act as tumor-suppressive structures in the skin [78].

KLK5 also contributes to epithelial barrier disruption in inflammatory and malignant conditions. Increased KLK5 expression has been observed in skin biopsies from patients with atopic dermatitis, together with reduced desmoglein-1 and filaggrin levels [79]. Consistently, KLK5 overexpression promoted desmoglein-1 and filaggrin degradation in bioengineered skin models, reproducing features of atopic dermatitis [79]. Beyond the skin, KLK5 silencing strengthened intercellular adhesion in oral epithelium, whereas KLK5-overexpressing tongue squamous carcinoma cells showed desmoglein degradation and loss of cell–cell cohesion [80]. Increased KLK5 activity has also been associated with epithelial barrier disruption and poor prognosis in eosinophilic esophagitis, gastric cancer, and cervical cancer [81,82,83,84,85].

**Figure 4:**
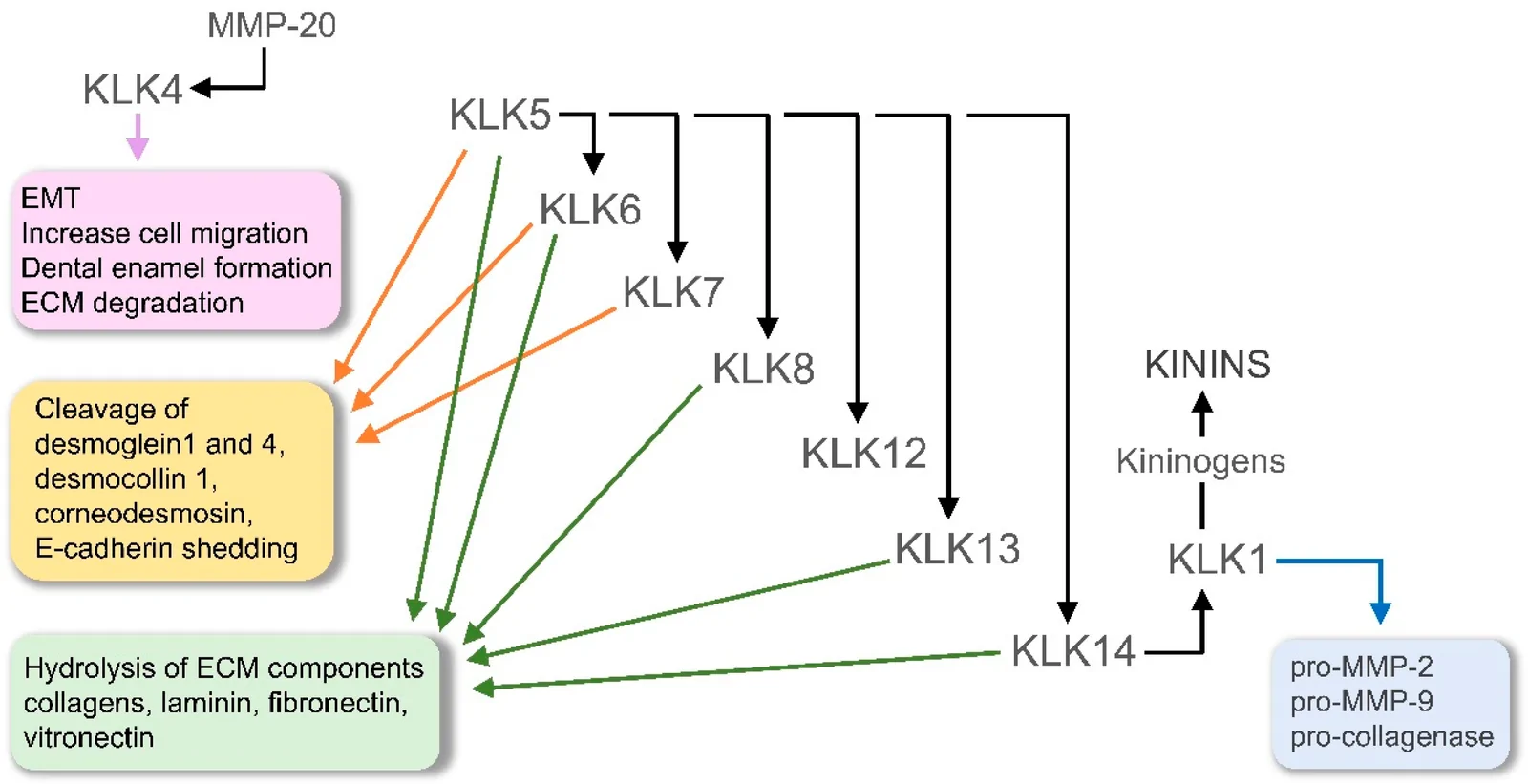
Schematic representation of the major KLKs involved in cell–ECM and cell–cell adhesion. KLK4 is activated by MMP-20, whereas KLK6, KLK7, KLK8, KLK12, KLK13, and KLK14 may be activated downstream of autoactivated KLK5. These KLKs participate in cell–cell adhesion remodeling, ECM degradation, and EMT-associated processes. KLK4 contributes to ECM remodeling by cleaving fibronectin, laminin, fibrin, and other ECM components, and by activating MMPs. KLK5–7 cleave intercellular adhesion molecules, destabilizing cell–cell junctions. KLK14 activates KLK1, the only KLK able to cleave kininogen precursors to generate kinins, thereby supporting a proinflammatory microenvironment. KLK1 may also activate pro-MMP-2, pro-MMP-9, and other collagenases, further promoting ECM remodeling. KLKs: kallikrein-related peptidases; MMPs: matrix metalloproteinases; EMT: epithelial–mesenchymal transition; ECM: extracellular matrix.

KLK6 has context-dependent roles in cancer biology. In mouse keratinocytes and HEK293 cells, KLK6 overexpression reduced membrane E-cadherin, increased nuclear β-catenin accumulation, and induced E-cadherin ectodomain shedding, thereby weakening intercellular cohesion [86]. Similar mechanisms have been described in gastric, colorectal, and pancreatic cancer models, where KLK6 promotes EMT-related signaling, ECM remodeling, and invasive behavior [87,88,89,90,91,92,93]. Clinically, elevated KLK6 expression correlates with enhanced motility and invasiveness in gastric cancer [91]. Conversely, in head and neck squamous carcinoma, KLK6 silencing promoted EMT-like changes and radioresistance in FaDu cells, whereas reduced KLK6 expression was associated with poorer patient survival [87]. Together, these findings indicate that KLK6 effects depend strongly on tissue type and tumor context (Figure 4).

KLK7 is one of the best-characterized KLKs involved in adhesion loss and tumor progression. In pancreatic carcinoma, KLK7 cleaves E-cadherin and desmogleins, disrupting adherens and desmosomal junctions and impairing cell aggregation in Panc-1 and BxPC-3 cells [94,95]. These effects favor invasive behavior and are supported by degradomic studies confirming KLK7-mediated cleavage of junctional proteins in epithelial cancers [94,96]. Beyond pancreatic tumors, elevated KLK7 expression has been associated with melanoma progression and metastasis, correlating with reduced E-cadherin, increased MCAM/CD146, and higher expression in metastatic lesions compared with primary tumors and benign nevi [97,98,99]. These findings support KLK7 as a regulator of epithelial cohesion and a potential biomarker of tumor dissemination (Figure 4). Recent evidence further supports this concept in pancreatic cancer. Eisenhauer et al. showed that KLK7 and KLK10 are upregulated in carcinoma in situ lesions and early-stage pancreatic ductal adenocarcinoma, where they are co-expressed with desmoglein-3. Importantly, desmoglein-3 was identified as a substrate for both KLK7 and KLK10, generating a 30-kDa extracellular fragment and suggesting that KLK-mediated desmosomal remodeling may occur early during pancreatic tumorigenesis [100]. This finding reinforces the role of KLKs as early modulators of desmosome-dependent cell–cell adhesion loss in cancer.

KLK4 also contributes to the regulation of epithelial adhesion and mesenchymal-like phenotypes. In gastric cancer cells, suppression of KLK4 by the long non-coding RNA LINC01314 reduced migration and Wnt/β-catenin signaling while increasing E-cadherin and decreasing N-cadherin levels [101]. Conversely, KLK4 overexpression in endometrial stromal cells was associated with enhanced migratory and angiogenic properties accompanied by reduced epithelial cohesion [102,103]. Consistently, coordinated overexpression of KLK4–7 decreased cell adhesion and integrin expression while promoting ECM remodeling [104,105].

In contrast to the pro-invasive effects reported for several KLKs, KLK13 appears to preserve epithelial cohesion in oral squamous cell carcinoma. Reduced KLK13 expression correlates with increased invasiveness, whereas KLK13 overexpression suppresses migration and upregulates multiple adherens and desmosomal junction proteins, including E-cadherin, catenins, desmogleins, and desmoplakin [106]. These findings support a tumor-suppressive role for KLK13 and highlight its potential as a prognostic biomarker in oral squamous cell carcinoma (Figure 4).

Altogether, KLKs regulate intercellular adhesion in cancer through effects on junctional proteins, epithelial integrity, and extracellular matrix remodeling. Depending on cellular context, individual KLKs may either promote or restrain invasive behavior, highlighting the complexity of KLK-regulated adhesive networks. These context-dependent functions support the potential of KLKs as biomarkers and therapeutic targets in tumor progression and dissemination (Figure 4).

## 3. Cell–ECM Adhesion: KKS Regulation of ECM Remodeling, Integrin Signaling, and Focal Adhesion Dynamics

Cell–ECM adhesion is coordinated at three interconnected levels—ECM composition, integrin engagement, and focal adhesion signaling—that together regulate cell attachment, cytoskeletal remodeling, migration, and transcriptional responses [68,107]. Integrins are the central mediators of this process: their extracellular domains bind ECM ligands such as collagen, laminin, fibronectin, and vitronectin, while their cytoplasmic tails recruit focal adhesion proteins including talin, paxillin, and vinculin, which connect to the actin cytoskeleton and activate FAK/Src-centered signaling cascades [108].

KKS components modulate each of these levels through complementary mechanisms: KLKs reshape the adhesive microenvironment by cleaving ECM proteins and activating MMPs, whereas kinin receptors B1R and B2R activate FAK/Src signaling, cytoskeletal remodeling, and integrin-dependent migration. Through these coordinated actions, the KKS contributes to ECM rearrangement, angiogenesis, inflammatory remodeling, and tumor dissemination [109].

### 3.1. Regulation of Integrin Engagement by KLKs, Kinin Receptors, and HKa

Among the integrin-regulatory mechanisms of the KKS, the roles of KLK1, B2R, and HKa in angiogenesis and cancer-associated adhesion have been most thoroughly characterized. Integrins connect ECM proteins to the actin cytoskeleton through focal adhesions, where adaptor and signaling proteins coordinate mechanical support and extracellular signal transduction [110,111,112,113,114,115,116], allowing cells to sense ECM composition and adjust adhesion strength, morphology, and intracellular signaling accordingly [2,3].

KLKs contribute to these processes by cleaving ECM components, releasing matrix-bound growth factors, and modifying focal adhesion environments. These mechanisms are increasingly recognized as drivers of tumor progression and metastasis [74]. During angiogenesis, integrin-dependent signaling supports endothelial cell migration and sprouting, contributing to tissue repair under physiological conditions and pathological neovascularization in tumors [117,118].

#### 3.1.1. Angiogenesis Regulation by KLK1, B2R, and HKa Through Integrin-Dependent Adhesion

KLK1 and B2R signaling promote angiogenesis through complementary effects on integrin expression, endothelial cell migration, and ECM remodeling. In endothelial progenitor cells (EPCs), KLK1 overexpression upregulates αvβ3 integrin and eNOS expression through PI3K/eNOS-dependent signaling, thereby enhancing angiogenic activity [119]. Since αvβ3 integrin mediates adhesion to RGD-containing ECM proteins and coordinates focal adhesion signaling, these findings suggest that KLK1 promotes angiogenesis by coupling integrin activation to ECM proteolysis [115,120,121]. In parallel, B2R activation in the same cell type drives MMP-2-dependent ECM remodeling, further supporting a pro-angiogenic role for the KLK1–kinin axis [110,119].

In contrast, cleaved HKa acts as an endogenous counter-regulator of this process. HKa inhibits αvβ3-dependent MMP-2 activation by reducing integrin ligand-binding affinity, thereby impairing ECM degradation and endothelial tube formation [122]. Together, these opposing activities indicate that KKS components can exert both pro- and anti-angiogenic effects depending on the balance between KLK1–B2R signaling and HKa-mediated suppression of αvβ3 integrin engagement. The anti-adhesive mechanisms of HKa are discussed in further detail in Section 3.5.

#### 3.1.2. Context-Dependent KLK Regulation of Integrin-Mediated Adhesion in Cancer

KLKs regulate integrin-dependent cell–ECM adhesion in a context-dependent manner, either enhancing adhesion and chemoresistance or promoting detachment and invasion through integrin downregulation and ECM remodeling [95,111,112,113,116,123,124,125,126,127,128]. These divergent effects likely depend on the integrin repertoire, ECM composition, protease inhibitor availability, and co-expression of other KLKs within the tumor microenvironment.

In ovarian carcinoma, KLK7 overexpression correlates with increased α5β1 integrin expression and enhanced adhesion to fibronectin and vitronectin, effects that are blocked by β1-integrin antibodies [111]. These cells also exhibit paclitaxel resistance and spheroid formation, suggesting that KLK7-mediated ECM–integrin interactions support peritoneal dissemination and survival in suspension. Similar chemoresistant phenotypes have been observed following overexpression of KLK4, KLK5, and KLK6 [111].

Conversely, coordinated overexpression of KLK4–7 reduces α5, αv, and β1 integrin expression and impairs adhesion to ECM substrates, supporting a role for KLKs in destabilizing cell–matrix interactions during tumor dissemination [112]. Likewise, KLK7 overexpression in melanoma and pancreatic cancer cells reduces adhesion to multiple ECM proteins, downregulates integrins involved in ECM recognition, and increases migratory and invasive behavior [95,97,114,127]. These effects are accompanied by increased MCAM/CD146 expression, reduced E-cadherin levels, and enhanced spheroid formation, features associated with metastatic progression [97,114].

In contrast, KLK5 and KLK8 may exert tumor-suppressive effects in specific contexts. In breast cancer cells, KLK5 overexpression reduces RhoA and MMP-9 activity and downregulates genes involved in ECM remodeling and adhesion signaling, correlating with reduced invasiveness and better prognosis [113,116,126]. Similarly, KLK8 overexpression in lung adenocarcinoma impairs fibronectin-mediated adhesion, reduces Src phosphorylation and migration-related signaling, and is associated with a less aggressive phenotype and favorable prognosis in early-stage NSCLC [123].

**Figure 5:**
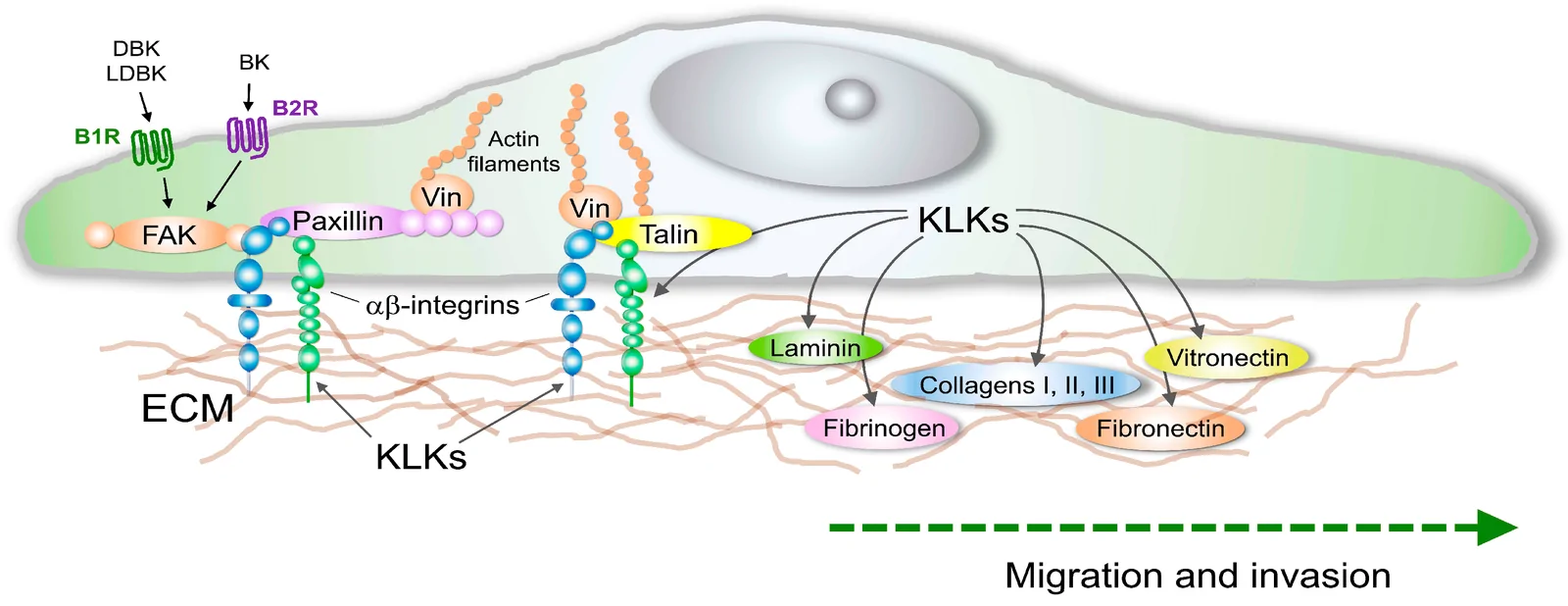
Regulation of cell–ECM interactions by KLKs and kinin receptors. KLK-mediated proteolysis may alter ECM ligand availability and integrin engagement by cleaving matrix components, including laminin, collagen, fibrinogen, fibronectin, and vitronectin. These changes can modulate integrin-associated focal adhesion complexes involving FAK, paxillin, talin, and vinculin, thereby influencing cytoskeletal organization, focal adhesion turnover, cell migration, and invasion. In parallel, kinin receptor activation, B1R by DBK/LDBK and B2R by BK, can induce FAK/Src phosphorylation, paxillin and p130Cas tyrosine phosphorylation, cytoskeletal remodeling, and adhesion-dependent cell motility. DBK: des[Arg^9^]-bradykinin; LDBK: Lys-des[Arg^9^]-bradykinin; BK: bradykinin; FAK: focal adhesion kinase; Vin: vinculin; ECM: extracellular matrix; KLKs: kallikrein-related peptidases; B1R: kinin B1 receptor; B2R: kinin B2 receptor.

Together, these findings indicate that KLKs dynamically regulate integrin signaling and cell–ECM adhesion, thereby influencing chemoresistance, spheroid formation, migration, and metastatic dissemination in a highly context-dependent manner (Figure 5).

### 3.2. Kinin Receptors Activate the FAK–Src Axis and Focal Adhesion Turnover

Kinin receptors regulate cell–ECM adhesion by activating focal adhesion signaling pathways. FAK is a non-receptor tyrosine kinase that modulates adhesion, movement, proliferation, and survival by controlling focal adhesion assembly and turnover [129,130]. Through interactions with integrins, Src kinases, paxillin, and p130Cas, FAK coordinates cytoskeletal remodeling and cell detachment from the ECM, both of which are required for migration and invasion [130,131]. B1R and B2R signaling directly activate the FAK/Src axis in structural and barrier-forming cells. In fibroblasts, keratinocytes, endothelial cells, vascular smooth muscle cells, and glioma cells, BK or LDBK stimulation induces phosphorylation of FAK, Src-family kinases, paxillin, and p130Cas, promoting focal adhesion remodeling and cell migration [41,132,133,134,135,136,137,138,139,140,141] (Figure 5).

Early studies in fibroblasts and HEK cells established that BK acting through B2R triggers tyrosine phosphorylation of FAK and p130Cas, linking kinin signaling to focal adhesion machinery [137]. Similar B2R-dependent activation of FAK/Src and downstream MAPK signaling has been described in vascular smooth muscle cells, fibroblasts, keratinocytes, and endothelial cells [41,132,134,135,137,138].

Kinin receptor-dependent FAK/Src activation also operates in pathological contexts of the central nervous system. In glioma cells, BK-induced FAK phosphorylation increases IL-8 expression and promotes cell migration through a STAT3-dependent mechanism mediated by B1R [140]. These findings suggest that B1R-driven focal adhesion signaling may contribute to glioblastoma invasiveness by coupling cytoskeletal remodeling to proinflammatory transcriptional responses.

Similar kinin receptor-dependent mechanisms have been described in epithelial cancers and during cutaneous repair. In breast cancer cell lines, kinin agonists increase migration and invasion through B1R- and B2R-dependent activation of the FAK/Src axis, effects that are abolished by pharmacological inhibition of either receptor or kinase [106]. LDBK also induces FAK phosphorylation and migration in prostate cancer cells, further supporting a pro-invasive role for B1R signaling in epithelial tumors [141]. Consistent with these findings, a recent review by Brusco and Oliveira emphasized that B1R and B2R participate not only in tumor progression but also in cancer-associated pain and anticancer therapy-induced pain, supporting their potential relevance as pharmacological targets in oncology [142].

Kinin signaling also affects cytoskeletal remodeling downstream of focal adhesion activation. In renal epithelial collecting duct cells, B2R activation by BK modulates the expression and redistribution of vinculin and talin, promoting focal adhesion remodeling, stress fiber formation, and actin reorganization through phosphatidylinositol 4,5-bisphosphate-dependent mechanisms [143,144]. In endothelial cells, B1R and B2R ligands induce filamin redistribution from the plasma membrane to the cytosol, in parallel with increased intracellular Ca^2+^ levels [145]. Since filamins organize actin networks and anchor signaling complexes to the cytoskeleton [146], these changes may contribute to altered cell shape and migratory behavior (Figure 5).

Similar cytoskeletal remodeling has been described in cancer models. In neuroblastoma cells, BK promotes F-actin polymerization and elongated actin filament formation, leading to increased cell spreading and surface occupancy [147]. Mechanistically, integrins such as β3 integrin participate in FAK/Src complex formation, stabilizing Src at the plasma membrane and promoting focal adhesion turnover [148]. In keratinocytes, B1R activation promotes adhesion-dependent motility relevant to wound healing and epidermal homeostasis [139]. Together, these findings indicate that kinin receptor-mediated focal adhesion remodeling is a conserved mechanism operating across diverse epithelial and mesenchymal cell types, with implications for both cancer progression and tissue repair (Figure 5).

### 3.3. Kinin Receptors and KLKs Regulate MMP-Dependent ECM Remodeling

Kinins contribute to cell adhesion and migration partly through the regulation of matrix metalloproteinases (MMPs), a family of zinc-dependent endopeptidases responsible for degrading ECM components, including collagens, laminin, elastin, and gelatin [149,150]. By modulating MMP expression and activity, kinin receptors influence ECM turnover, basement membrane disruption, and cell invasion.

Our group demonstrated that LDBK stimulates MMP-2 and MMP-9 expression and release through ERK1/2-dependent signaling in breast cancer cells [149]. Increased gelatinase activity was associated with distinct invasive profiles, with MCF-7 cells showing predominant MMP-2 activity and MDA-MB-231 cells exhibiting higher MMP-9 activity [149]. Since both gelatinases degrade basement membrane components such as laminin and type IV collagen, their induction may facilitate tumor invasion and ECM remodeling [151].

Similar effects have been reported in neuroblastoma models, where BK increases MMP-2 expression and gelatinase activity, correlating with enhanced proliferative behavior [147]. This response is attenuated by pharmacological inhibition of the P2X7 purinergic receptor, suggesting functional crosstalk between kinin and purinergic signaling pathways during tumor progression and bone marrow metastasis [147]. One hypothesis is that this crosstalk may involve BK-induced ATP release and Ca^2+^ mobilization, leading to P2X7-dependent amplification of ERK/MMP signaling and thereby promoting ECM remodeling, migration, and metastatic behavior.

KLKs may also converge on MMP-dependent remodeling. In pancreatic ductal carcinoma, KLK10 overexpression promotes EMT, migration, invasion, and activation of the FAK/Src/ERK cascade, whereas KLK10 silencing reduces phosphorylation of FAK, Src, and ERK and decreases metastatic dissemination in vivo [152]. Together, these findings position KKS components as regulators of MMP-dependent ECM remodeling, focal adhesion signaling, and cell migration in cancer and tissue remodeling.

### 3.4. Direct KLK Cleavage of ECM Proteins in Cancer and Angiogenesis

Beyond MMP regulation, KLKs directly remodel the ECM by cleaving matrix components and modulating the adhesive landscape [153,154,155]. KLKs cleave ECM proteins including collagens, fibronectin, laminin, gelatin, and casein, thereby altering cell–matrix adhesion, tissue architecture, and migratory behavior.

In estrogen-sensitive breast cancer, activation of the KLK–kinin axis through B1R signaling promotes tumor progression by coordinately regulating multiple KLKs. LDBK-induced upregulation of KLK6 enhances invasive behavior through ECM degradation, whereas increased KLK11 expression promotes proliferation and potentially angiogenic signaling through IGFBP-3 degradation and subsequent IGF-1 release. Concurrent downregulation of the tumor suppressor KLK10 further favors malignant behavior [154]. Similarly, KLK6 silencing in colon cancer cells increases the accumulation of ECM proteins, including collagens, fibronectin, laminin, and vitronectin, supporting a direct role for KLK6 in ECM turnover and invasiveness [156].

KLK-mediated ECM remodeling also contributes to physiological and pathological angiogenesis. KLK1 cleaves fibronectin and collagen type IV and activates tissue plasminogen activator (tPA), promoting ECM degradation and tissue remodeling in the uterine endometrium during the proliferative phase [155]. Likewise, KLK12 regulates angiogenesis by cleaving fibronectin and tenascin-C, impairing ECM fibril assembly and reducing endothelial cell adhesion, thereby enhancing migratory behavior [153]. Importantly, blockade of fibronectin proteolysis abolishes these effects, highlighting the functional relevance of KLK12-mediated ECM remodeling during neovascularization [153].

Overall, KLKs regulate tissue barriers in cancer and angiogenesis by degrading ECM components and reshaping cell–matrix adhesion. This proteolytic activity cleaves key proteins such as collagen and fibronectin, promotes tumor invasion, and modulates endothelial adhesion during angiogenic remodeling (Figure 5).

### 3.5. HKa as an Endogenous Anti-Adhesive Regulator

HKa is an endogenous regulator of cell–ECM interactions with prominent anti-adhesive and anti-angiogenic properties [157,158]. HKa contains a histidine-rich D5 domain that binds ECM proteins such as vitronectin, thereby interfering with integrin-dependent adhesion and endothelial cell attachment [159,160,161,162,163].

Functional studies have shown that HKa impairs endothelial cell adhesion to vitronectin- and gelatin-coated matrices while reducing FAK and paxillin phosphorylation, indicating suppression of focal adhesion signaling [157]. HKa also disrupts the interaction between urokinase plasminogen activator receptor (uPAR) and vitronectin in a Zn^2+^-dependent manner, further supporting its role in regulating integrin-associated adhesion pathways [163].

**Figure 6:**
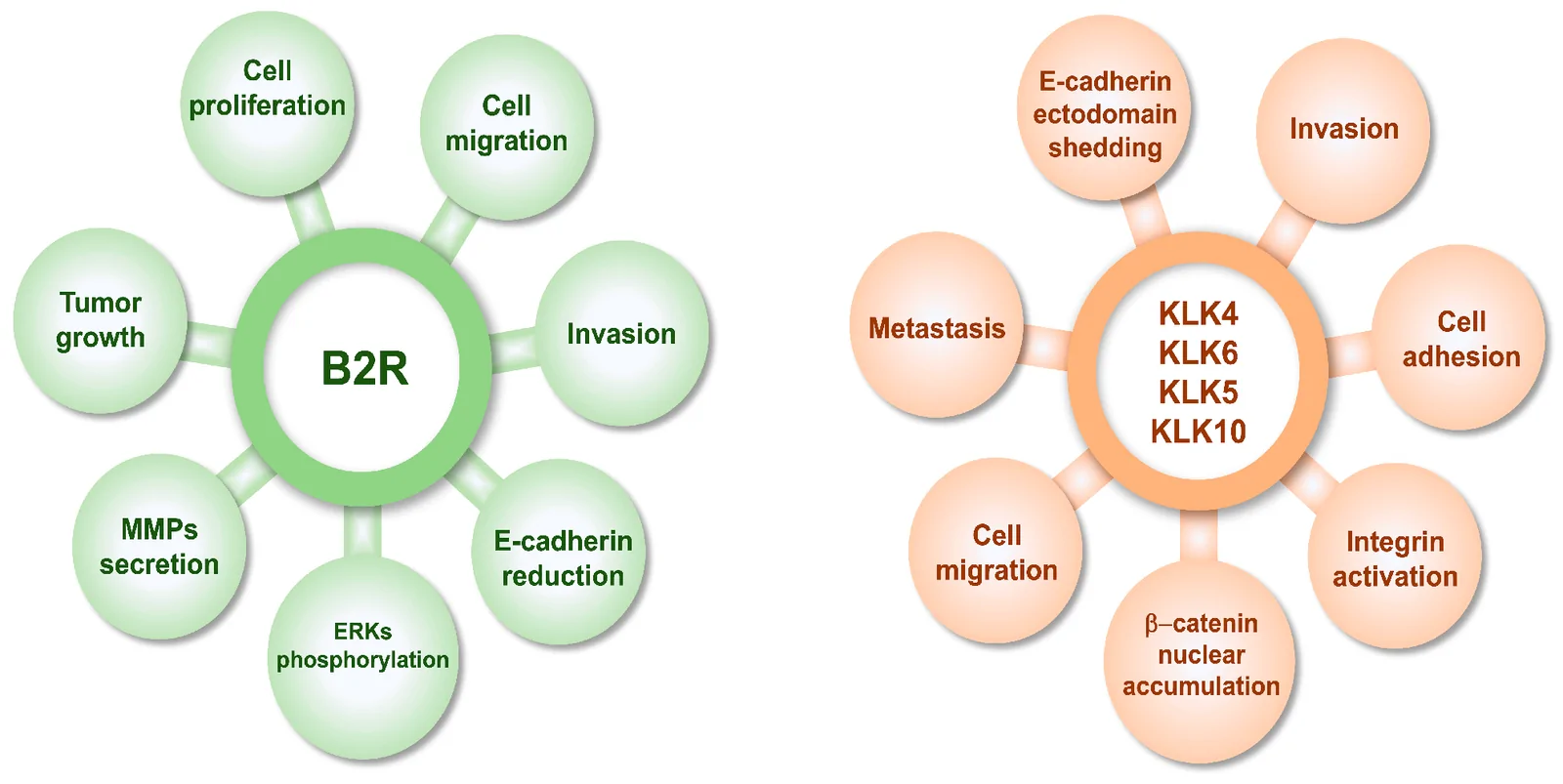
Role of B2R and KLKs in tumorigenesis. Activation of B2R by bradykinin (BK), shown on the left, and increased activity of selected KLKs, including KLK4, KLK5, KLK6, and KLK10, shown on the right, promote key tumor-associated processes such as proliferation, migration, invasion, integrin activation, MMP/ERK signaling, and tumor progression. B2R: kinin B2 receptor; MMPs: matrix metalloproteinases; ERKs: extracellular signal-regulated kinases; KLKs: kallikrein-related peptidases.

Altogether, these findings identify HKa as an endogenous inhibitor of integrin-mediated adhesion and focal adhesion signaling, with important implications for angiogenesis, vascular remodeling, inflammation, and cancer-associated migration. The mechanisms described in Section 3 highlight the dual, context-dependent capacity of the KKS to promote or restrain tumor-associated adhesion remodeling, as summarized in Figure 6.

## 4. Challenge and Future Perspectives

Despite strong experimental evidence linking the KKS and KLK family members to adhesion remodeling, inflammation, fibrosis, and cancer progression, clinical translation remains limited. The main challenge is a translational paradox: the same pathways that drive pathological adhesion, ECM remodeling, leukocyte recruitment, and tumor dissemination also participate in physiological processes such as immune surveillance, vascular homeostasis, wound healing, and tissue repair [164,165]. This complexity is further supported by recent evidence showing that epithelial KLK1 may restrain colorectal tumorigenesis by suppressing B1R-mediated fibroblast phenotypic transition, highlighting that KKS/KLK signaling cannot be uniformly interpreted as pro-tumorigenic across all tissues and disease stages [166]. This functional duality limits the development of broad therapeutic strategies and highlights the need for context-specific targeting. In this context, several mechanistic gaps still need to be addressed. First, quantitative in vivo data are scarce regarding how KLK activity modifies integrin expression or affinity, ECM ligand availability, focal adhesion turnover, and cytoskeletal remodeling. Second, the crosstalk between kinin receptors, CAMs, integrins, MMPs, KLKs, and focal adhesion proteins remains incompletely defined across different stromal, immune, endothelial, and tumor cell populations. Third, the temporal dimension of KKS signaling, acute versus chronic activation, remains insufficiently integrated into therapeutic design. Addressing these gaps will be essential to translate experimental findings into clinically meaningful interventions.

Although kinin receptor antagonists and KLK-targeting strategies have shown anti-inflammatory, anti-invasive, and anti-metastatic effects in experimental models, robust clinical evidence remains limited [167]. Recent biomaterial-based pancreatic cancer models also suggest that KLK6 may act as a mediator of neutrophil recruitment, immunosuppression, and response to anti-PD-1 therapy, reinforcing the need to evaluate KLKs not only as regulators of adhesion and invasion, but also as modulators of tumor immune remodeling [45]. In this regard, recent advances in KLK-directed drug discovery, including activity-based probes, selective inhibitors, substrate-guided approaches, and targeted prodrug strategies, provide a framework for developing more precise interventions against disease-relevant KLK activities [168].

Future studies should prioritize selective modulation rather than broad inhibition. Promising strategies may include receptor-specific targeting, tissue- or disease-stage-restricted approaches, combination therapies with ECM- or integrin-directed agents, and biomarker-guided patient stratification based on receptor expression, KLK activity, and inflammatory status. Beyond oncology, KKS- and KLK-mediated adhesion remodeling is relevant to chronic inflammatory and fibrotic diseases, including arthritis, asthma, liver fibrosis, atopic dermatitis, vascular remodeling, and atherosclerosis. These conditions reinforce the broad biological relevance of the pathway but also emphasize the safety concerns associated with systemic inhibition.

In conclusion, the KKS/KLK network represents a biologically attractive but therapeutically complex target. Progress in the field will require moving from descriptive associations toward quantitative, cell-specific, and temporally resolved models of adhesion remodeling. Such advances will be essential to dissociate pathological adhesion and ECM remodeling from physiological tissue repair and immune defense.

## Data Availability

No new data were created or analyzed in this study.

## Abbreviations

The following abbreviations are used in this manuscript:

KKS	Kallikrein–Kinin system
BK	Bradykinin
ECM	Extracellular matrix
EMT	Epithelial–mesenchymal transition
cell–ECM	Cell–extracellular matrix
KLKs	Kallikrein-related peptidases
B2R	Kinin B2 receptor
LDBK	Lys-des[Arg^9^]-bradykinin
DBK	Des[Arg^9^]-bradykinin
B1R	Kinin B1 receptor
FAK	Focal adhesion kinase
TNF-α	Tumor necrosis factor-alpha
IL-1β	Interleukin-1 beta
IFN-γ	Interferon-gamma
GPCR	G protein-coupled receptor
LTB4	Leukotriene B4
MCP-1	Monocyte chemoattractant protein-1
PMNs	Polymorphonuclear leucocytes
Mac-1	Macrophage-1 antigen
HMEC-1	Human microvascular endothelial cell line-1
ICAM-1	Intercellular adhesion molecule type 1
LFA-1	Lymphocyte function-associated antigen-1
LL-37	Cathelicidin
MMP-9	Matrix metalloproteinase-9
NO2Tyr	Nitrotyrosine
PECAM-1	Platelet endothelial cell adhesion molecule-1
ELAM-1	Endothelial leukocyte adhesion molecule-1
HUVEC	Human umbilical vein endothelial cells
MAPK	Mitogen-activated protein kinase
NF-κB	Nuclear factor kappa-light-chain-enhancer of activated B cells
LPS	Lipopolysaccharides
PLC	Phospholipase C
PKC	Protein kinase C
ICAM-2	Intercellular adhesion molecule-2
RAGE	Receptor for advanced glycation end products
JAM	Junctional adhesion molecule
D2R	Dopamine receptor type 2
STAT3	Signal transducer and activator of transcription 3
ADAM	Disintegrin and Metalloproteinase
ANCA	Anti-neutrophil cytoplasmic antibodies
CAMs	Cellular adhesion molecules
α-SMA	Alpha-smooth muscle actin
PDGF	Platelet-derived growth factor
CTGF	Connective tissue growth factor
VEGF	Vascular endothelial growth factor
NCA	Neutrophil chemotactic activity
MCA	Monocyte chemotactic activity
IL-6	Interleukin-6
IL-8	Interleukin-8
G-CSF	Granulocyte-colony stimulating factor
TGF-β	Transforming growth factor-beta
PI3K	Phosphoinositide 3-kinase
PKB/Akt	Protein Kinase B
Rac1	Ras-related C3 botulinum toxin substrate 1
ZO-1	Zonula occludens-1
MMP	Matrix metalloproteinase
TPA	12-O-tetradecanoylphorbol-13-acetate
hKLK7	Human Kallikrein 7
MCAM	Melanoma Cell Adhesion Molecule
CD146	Cluster of Differentiation 146
lncRNA	Long non-coding RNA
siRNA	Small interfering RNA
Grb2	Growth Factor receptor-bound protein 2
Src	Proto-oncogene protein kinase c-Src
eNOS	Endothelial Nitric Oxide Synthase
RGD	Arginyl glycyl aspartic acid
HKa	Cleaved high-molecular-weight kininogen
GTPase	Guanosine Triphosphate hydrolase
ITGB1	β1-integrin gene
EZR	Ezrin
ITGB5	β5-integrin
COL12A1	Collagen type XII alpha 1 chain
COL24A1	Collagen type XXIV alpha 1 chain
PIP2	Phosphatidylinositol 4,5-bisphosphate
p130Cas	Src transformation-associated protein p130
ATP	Adenosine triphosphate
P2X7	P2X purinoceptor 7
IGFBP-3	Insulin-like growth factor-binding protein 3
IGF-I	Insulin-like growth factor I
tPA	Tissue plasminogen activator
E2	Estradiol-17-beta
PtdIns(4,5)P2	Phosphatidylinositol 4,5-bisphosphate
HK	High-molecular-weight kininogen
uPAR	Urokinase plasminogen activator surface receptor
